# Swimming capability of zebrafish is governed by water temperature, caudal fin length and genetic background

**DOI:** 10.1038/s41598-019-52592-w

**Published:** 2019-11-08

**Authors:** Yuma Wakamatsu, Kazutoyo Ogino, Hiromi Hirata

**Affiliations:** Department of Chemistry and Biological Science, College of Science and Engineering, Aoyama Gakuin University, Sagamihara, 252-5258 Japan

**Keywords:** Molecular biology, Zoology

## Abstract

Several zebrafish strains such as AB, Tübingen (TU), Wild India Kolkata (WIK) and Tupfel long fin (TL) have been established for genetic study. Each strain has its morphological and behavioral traits. Motor traits, however, have not been explored in zebrafish strains. We here applied a treadmill for fish (swimmill) and measured swimming capability of adult zebrafish by critical swimming speed, which is the maximum water velocity in which fish can keep swimming. First, we confirmed that swimming capability does not vary between female and male. Second, we found that the appropriate water temperature for swimming was between 16 and 30 °C. Third, our fin clip experiments using long-finned zebrafish revealed that they can exhibit high swimming capability when the caudal fin length was set between 3 and 10 mm, implying that long-finned zebrafish are unfavorable for fast swimming. Finally, we compared swimming capability of several zebrafish strains and demonstrated that WIK fish was significantly less capable of swimming despite that they have short caudal fin (~9 mm). The offspring of WIK fish were less capable of swimming, while hybrids of WIK and TU showed high swimming performance comparable to TU. Thus, lower swimming capability of WIK strain is inheritable as a motor trait.

## Introduction

Swimming capability is a motor characteristics of fish species. To quantitatively analyze fish swimming, a treadmill for fish (swimmill) such as Swim tunnel respirometer of Loligo Systems^1^ and flume^2^ has been developed. These equipments generate a directional water flow at certain velocity in a small chamber using a spinning propeller or pump. By increasing the water velocity in a stepwise manner, one can measure the maximum swimming speed (*U*_max_), which is also referred to as critical swimming speed (*U*_crit_). Since *U*_crit_ is measurable in any fish and is different by fish species^3^, *U*_crit_ is a good readout for swimming capability. Previous studies by Conradsen demonstrated that *U*_crit_ of male zebrafish (*Danio rerio*) is higher than that of female zebrafish^1,4^. On the contrary, another report suggested that swimming capability of female and male zebrafish was comparable if they were reared at 25~28 °C, which is the standard condition of zebrafish maintenance in laboratories^5^. This contradiction of swimming performance between genders has been unsolved. Several investigations has also focused on water temperature during swimming and revealed that swimming capability of large fish such as largemouth bass (*Micropterus salmoides*) and rainbow trout (*Oncorhynchus mykiss*) was compromised at low and high temperatures^3,6^. In the case of zebrafish, *U*_crit_ has not been assayed at low and high temperatures. Plaut assessed the length of caudal fin in swimming and found that swimming capability of no-tail zebrafish, which carry a homozygous mutation in T-box gene and thus completely lack caudal fin, is 65% lower than that of wild-type that have 7.1~9.6 mm caudal fin^7^. Interestingly, long-finned zebrafish that have 11.2~24.8 mm caudal fin showed 22% lower swimming performance compared to wild-type^7^. These results suggest that zebrafish have adequate length of caudal fin, neither too long nor too short, for swimming.

A variety of wild type strains of zebrafish including AB, Tübingen (TU), Wild India Kolkata (WIK) and Tupfel long fin (TL), has been developed for genetic study. The most popular AB strain was originated from two lines, A and B, each purchased from a pet shop in Oregon at different times and has been maintained since 1970s^8^. The TU stain derived from a pet store has been maintained by inbred breeding in Tübingen, Germany and used for genome sequencing project in Sanger Institute^8^. The WIK stain originated from a single pair mating of wild zebrafish caught at Kolkata, India has been kept with a high level of genetic heterogeneity^8^. The TL strain carries homozygous *leo/gja5b* and unidentified *lof* mutations, causing spotting pigment pattern and long fins, respectively^8^. In addition to these morphological differences, TL strain exhibits physiological and behavioral differences. For example, TL fish show lower hypothalamus-pituitary-internal cells (HPI) axis function compared to AB fish in both larval and adult stages^9^. The reduction of escape probability to repetitive exposure to acoustic/vibrational stimuli, which is referred to as habituation, is less markedly seen in TL^10^. The other report showed that basal blood glucose level in WIK fish is lower than that in TU fish^11^. These evidence with many others indicates that different strains have different genetic traits. However, whether these traits are inherited to the offspring has been largely unclear.

In this study, we applied the Swim tunnel respirometer to quantitatively analyze *U*_crit_ of adult zebrafish and confirmed that there is no gender difference in swimming capability and that zebrafish can swim well at 16–30 °C water temperature. Our swimming assay using fin-clipped zebrafish revealed that caudal fin length is associated with swimming capability and that 3 mm length of caudal fin is sufficient to fulfill high swimming performance. We also demonstrated that WIK is less capable of swimming compared to the other wild-type strains and that this reduced motor trait is ameliorated in the next generation by outcrossing with TU stain, suggesting a genetic component of swimming capability.

## Results

### Swimming capability of zebrafish is not affected by gender

**Figure 1:**
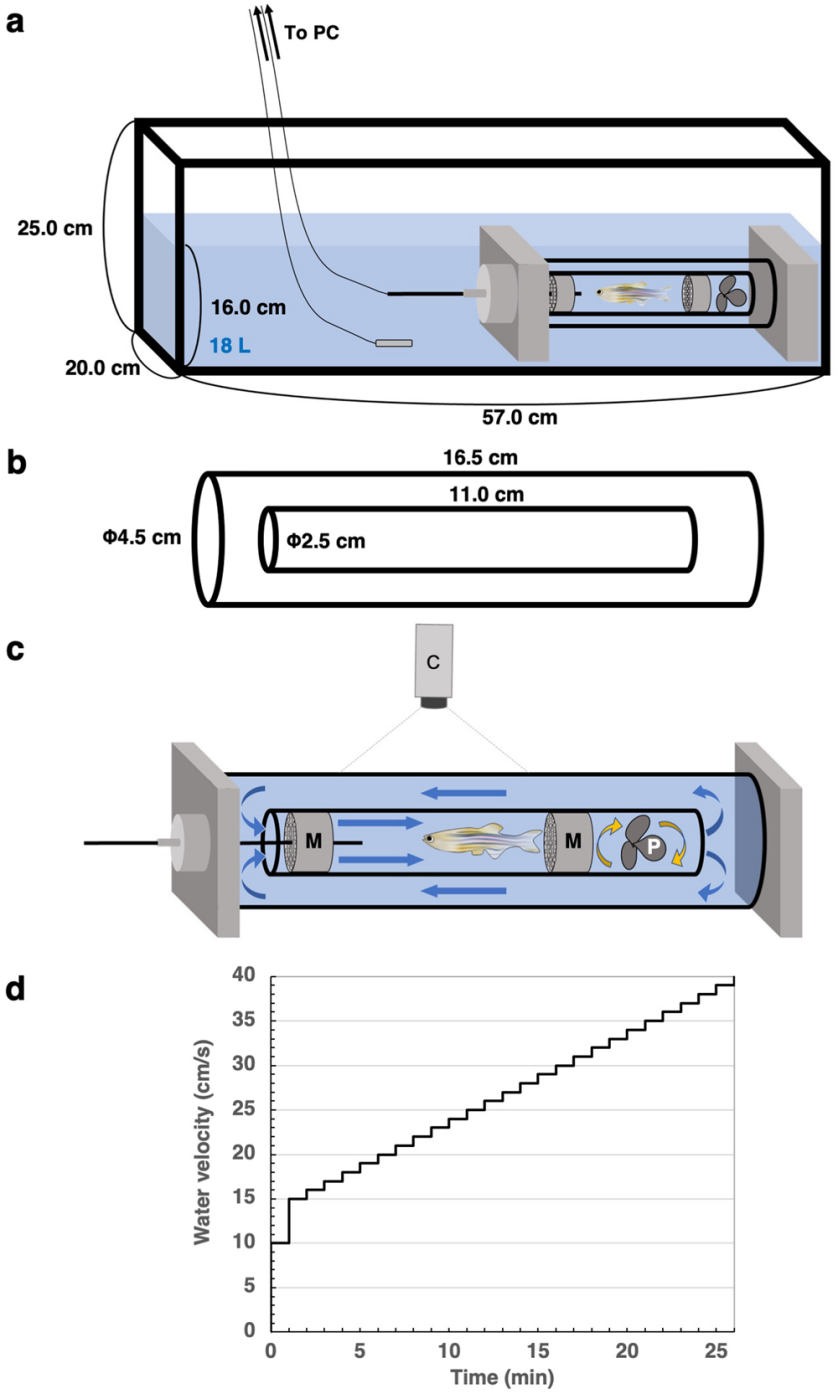
A scheme of the swimming assay. (**a**) The whole view of the swimmill equipment used in this study. (**b**) The size of the cylinder chambers. (**c**) The enlarged view of the swimming chamber. Zebrafish swim in the water flow to stay at the same position. Two plastic meshes were put in the small cylinder to avoid the fatigued fish hit to the spinning propeller. M: mesh; P: propeller; C: high speed camera; Blue arrow: water flow. (**d**) Protocol of incremental changes in water velocity.

To investigate swimming capability of adult zebrafish, we applied a swim tunnel respirometer, which is a swimmill system. A zebrafish was put under a propeller-driven water flow in a chamber, and fish was compelled to swim in the water flow (Fig. 1a–c). The water velocity increased 1 cm/s every 1 min after initial warming up of 10 cm/s flow for 1 min and successive 15 cm/s flow for 1 min (Fig. 1d). Zebrafish swam at the speed of water flow until the water flow went above the swimming capability of the fish. The water velocity at when zebrafish can no longer keep swimming was defined as critical swimming speed (*U*_crit_) as described previously^12^.

**Figure 2:**
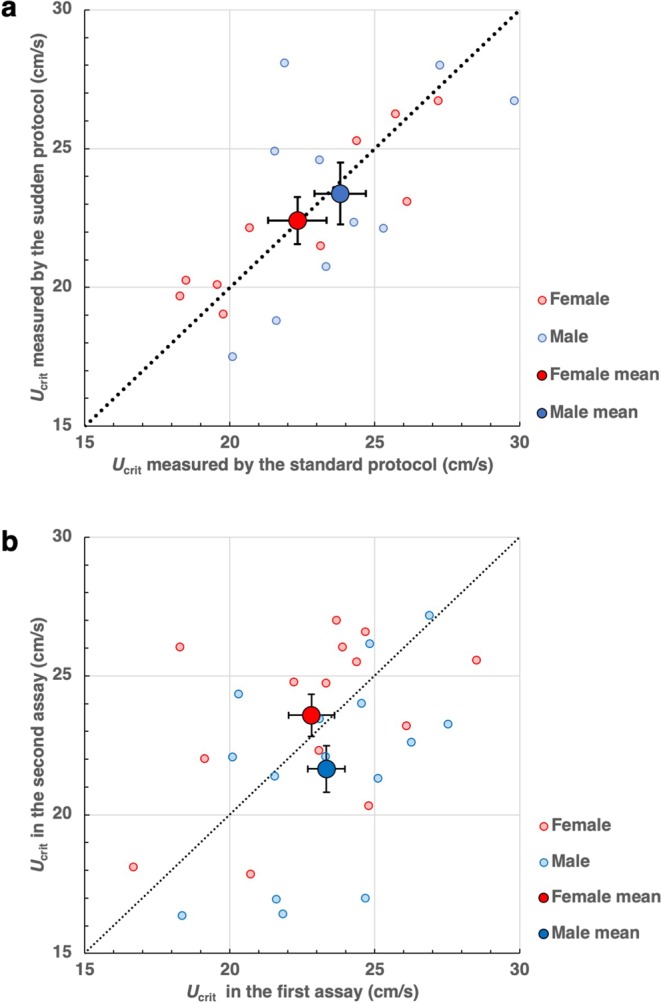
The swimming capability of zebrafish is not affected by gender. (**a**) The *U*_crit_ of PET strain fish was measured for two successive days using the standard protocol in the first day and sudden swimming protocol in the second day. (**b**) The *U*_crit_ of PET strain fish was measured for two successive days using the same standard protocol. Data from female fish (red) and male fish (blue) are displayed in the same graph. Big red and blue circles show mean *U*_crit_ of female and male, respectively. Bars on the big circles show sem.

Former studies have raised an argument on whether swimming performance of adult zebrafish vary between female and male^1,4,5^. To address this contradiction, we measured *U*_crit_ of adult long-finned zebrafish, which were purchased from a local pet store and thus referred to as PET strain. The *U*_crit_ of female was 22.3 ± 1.0 cm/s (n = 10), while that of male was 23.8 ± 0.9 cm/s (n = 10; P > 0.3). To check whether our standard protocol evaluates the maximum swimming capability under no influence of fatigue after 10 min (108.6 m) of swimming, we tested a different protocol, in which the water velocity starting at (*U*_crit_-3) cm/s followed by 1 cm/s increase every 1 min. In this sudden swimming protocol, the *U*_crit_ of female was 22.4 ± 0.8 cm/s (n = 10), and that of male was 23.4 ± 1.1 cm/s (n = 10; P > 0.3). In both female and male, the *U*_crit_ was comparable between two protocols (P > 0.3; Fig. 2a). Hence, *U*_crit_ evaluated by our standard protocol shows maximal swimming capability of zebrafish. To further check whether *U*_crit_ is reproducible, we measured *U*_crit_ on two successive days using the same standard protocol starting from 10 cm/s water velocity. The *U*_crit_ of female (n = 14) was 22.8 ± 0.8 cm/s on the first day and 23.6 ± 0.8 cm/s on the second day, while the *U*_crit_ of male (n = 15) was 23.3 ± 0.6 cm/s on the first day and 21.6 ± 0.8 cm/s on the second day (Fig. 2b). The *U*_crit_ was comparable between the first and the second days in both female (P > 0.1) and male (P > 0.1). More importantly, *U*_crit_ of female and male were comparable (P > 0.1) in our standard protocols. Thus, we hereafter show the *U*_crit_ of the second day without distinction of female and male.

### Adequate water temperature for zebrafish swimming is from 16 °C to 30 °C

**Figure 3:**
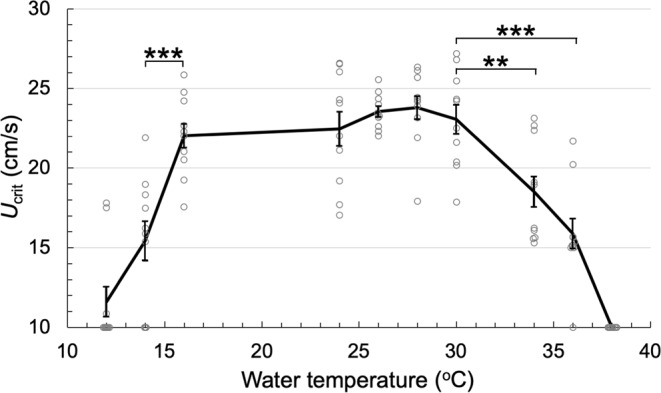
Water temperature adequate for zebrafish swimming is between 16 and 30 °C. The *U*_crit_ of PET strain fish were measured at different water temperatures from 12 to 38 °C. The *U*_crit_ in ranged from 16 to 30 °C were comparable. The *U*_crit_ at 12 and 14 °C were significantly lower than that at 16 °C. The *U*_crit_ at 34, 36 and 38 °C were also significantly lower than that at 30 °C. Values are mean ± sem. The P values are calculated using Student’s t test. ^**^P < 0.01; ***P < 0.001.

To assess the effect of water temperature in swimming capability, we measured the *U*_crit_ of zebrafish PET strain at different water temperature from 12 to 38 °C (Fig. 3). The *U*_crit_ of PET strain was comparable between 16 and 30 °C (16 °C: 22.0 ± 0.8 cm/s, n = 10; 24 °C: 22.5 ± 1.1 cm/s, n = 10; 26 °C: 23.6 ± 0.3 cm/s, n = 10; 28 °C:  23.8 ± 0.7 cm/s, n = 10; 30 °C: 23.1 ± 0.9 cm/s, n = 10). At lower water temperature below 16 °C, the *U*_crit_ of PET strain was significantly lower (14 °C: 15.4 ± 1.3 cm/s, n = 10; 12 °C:  11.6± 1.0 cm/s, n = 10) than normal. Some fish failed to swim in 10 cm/s water velocity during initial warming up. Similarly, the *U*_crit_ of PET strain was lower at high water temperature above 30 °C (34 °C: 18.5 ± 1.0 cm/s, n = 10; 36 °C: 15.9 ± 0.9 cm/s, n = 10). Furthermore, all zebrafish failed to swim in 10 cm/s water velocity at 38 °C. These results indicate that the adequate water temperature for zebrafish swimming is between 16 and 30 °C.

### Caudal fin of 3 mm length is sufficient for zebrafish swimming

**Figure 4:**
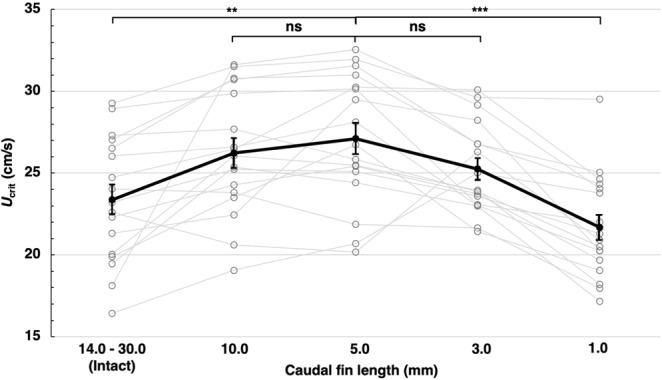
The length of caudal fin adequate for zebrafish swimming is between 3 and 10 mm. The *U*_crit_ of intact long-finned PET strain were measured. They were subsequently fin-clipped and subjected to *U*_crit_ measurement. The *U*_crit_ of fin-clipped fish with the caudal fin ranged from 3 to 10 mm were comparable. The *U*_crit_ of intact finned (14~30 mm) zebrafish was significantly lower than that of 5 mm caudal fin fish. Similarly, the *U*_crit_ of 1 mm caudal fin zebrafish were significantly lower than that of 5 mm caudal fin fish. Values are mean ± sem. The P values are calculated using Student’s t test. ^**^P < 0.01; ^***^P < 0.001; ns: not significant.

A previous study suggested that long-finned zebrafish is less capable of swimming compared to short-finned zebrafish^7^. To detail the length of caudal fin in swimming capability, we measured the *U*_crit_ of long-finned zebrafish PET strain and repeated the measurement after clipping the fin by degrees (Fig. 4). The *U*_crit_ of PET strain with intact 14~30 mm caudal fin was 23.4 ± 0.9 cm/s (n = 17). The caudal fin of these zebrafish was shortened to 10 mm length and fin-clipped fish were assayed for swimming capability. The *U*_crit_ of 10 mm caudal fin fish was 26.2 ± 0.9 cm/s. Interestingly, the *U*_crit_ of 5 mm caudal fin fish was 27.1 ± 1.0 cm/s and was significantly higher than that of intact long-finned fish (P < 0.01). Zebrafish that have only 3 mm caudal fin showed normal swimming performance (*U*_crit_: 25.2 ± 0.7 cm/s), whereas 1 mm caudal fin fish showed significantly lower swimming capability (*U*_crit_: 21.7 ± 0.8 cm/s, P < 0.001). Collectively, zebrafish can swim normally with a caudal fin ranged from 3 to 10 mm length.

### WIK strain is less capable of swimming

**Figure 5:**
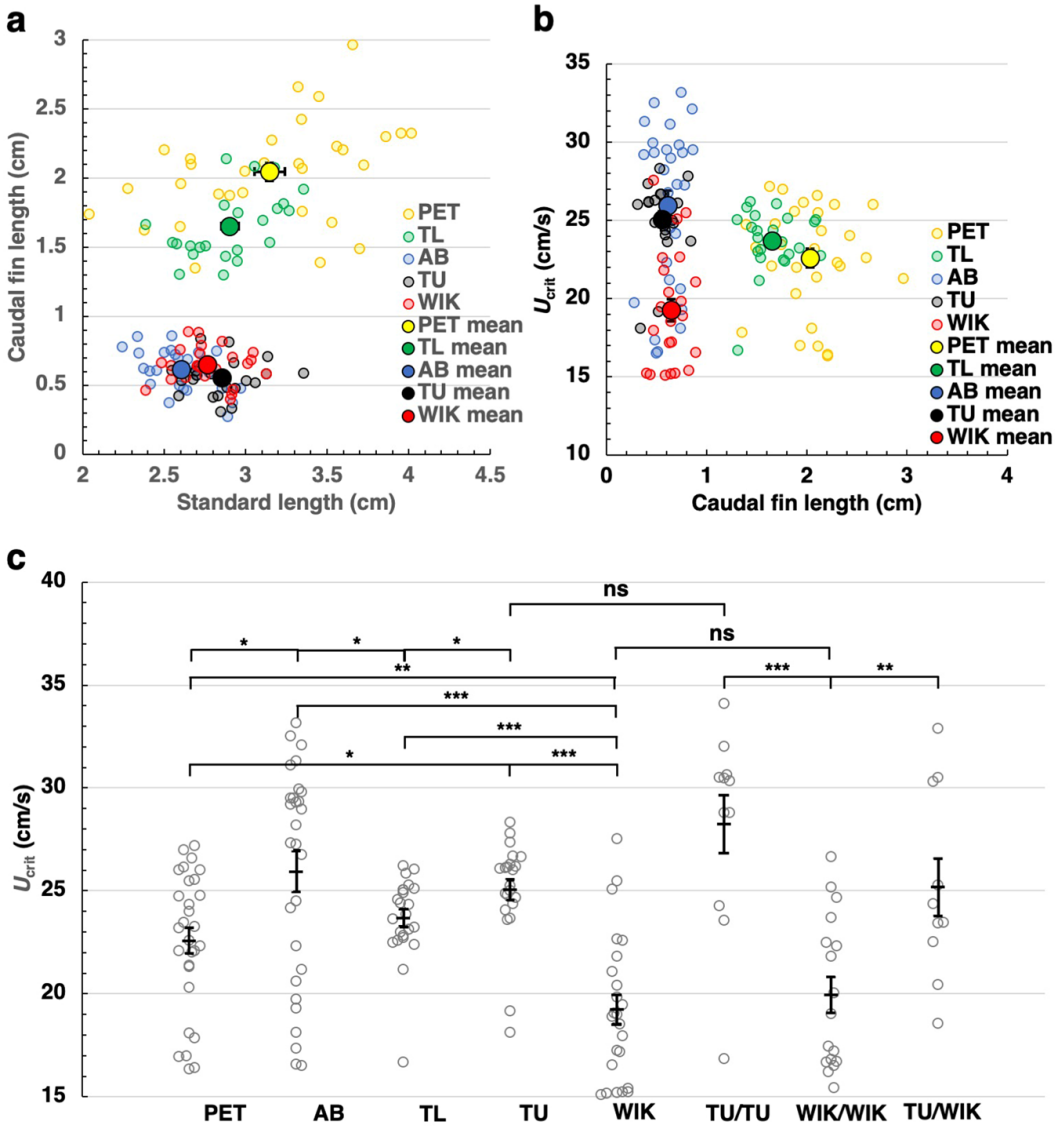
WIK is less capable of swimming, and the motor trait is inherited to offspring. (**a**) The SL and CFL of PET, TL, AB, TU and WIK fish were measured. While PET and TL had a long fin (13~mm), AB, TU and WIK had a short fin (~9 mm). (**b**) The *U*_crit_ of five zebrafish strains were measured. (**c**) A graph showing the *U*_crit_ of five zebrafish strains and their offsprings. The *U*_crit_ of AB and TU were comparable. The *U*_crit_ of PET and TL strains were significantly lower than that of AB and TU. The *U*_crit_ of WIK strain was significantly lower than that of PET and TL. The TU/TU and TU/WIK offsprings showed normal swimming capability. The *U*_crit_ of WIK/WIK offspring was significantly lower than that of TU/TU and TU/WIK. Values are mean ± sem. The P values are calculated using Student’s t test. ^*^P < 0.05; ^**^P < 0.01; ^***^P < 0.001; ns: not significant.

To compare the swimming capability between different zebrafish strains, we obtained wild-type zebrafish (AB, TL, TU and WIK) from zebrafish stock center ZIRC. We measured caudal fin length (CFL) along with standard length (SL), which is the length from head to the root of the caudal fin. We found that these four strains and PET strain can be classified into two groups by CFL. AB, TU and WIK have short fin (AB: 6.1 ± 0.3 mm, n = 28; TU: 5.6 ± 0.3 mm, n = 22; WIK: 6.5 ± 0.3 mm, n = 24), while PET and TL have long fin (PET: 20.0 ± 0.6 mm, n = 29; TL: 16.5 ± 0.5 mm, n = 23; Fig. 5a). We then measured *U*_crit_ of these fish (Fig. 5b,c). The *U*_crit_ of AB and TU were comparable (AB: 25.9 ± 1.0 cm/s; TU: 25.1 ± 0.5 cm/s; P > 0.3). In agreement with our finding that long-finned zebrafish were less capable of swimming, *U*_crit_ of PET and TL strain was significantly lower (PET: 22.6 ± 0.6 cm/s; TL: 23.6 ± 0.4 cm/s) than that of AB and TU. Unexpectedly, the *U*_crit_ of WIK strain was significantly lower (19.3 ± 0.8 cm/s, n = 24) than that of PET and TL strains, despite that WIK fish have a short fin.

To further address whether this low capability of swimming in WIK is a heritable trait, we raised zebrafish offspring generated by crossing TU and TU (TU/TU), WIK and WIK (WIK/WIK) and TU and WIK (TU/WIK) pairs, and measured *U*_crit_ for each offspring. As expected, the *U*_crit_ of TU/TU (28.2 ± 1.4 cm/s, n = 11) was comparable to that of originally purchased TU. On the other hand, the *U*_crit_ of WIK/WIK (19.9 ± 0.9 cm/s, n = 17) was significantly lower than that of TU/TU and was comparable to that of originally purchased WIK. In addition, the *U*_crit_ of TU/WIK (25.2 ± 1.4 cm/s, n = 10) was significantly higher than that of WIK/WIK and was comparable to that of originally purchased TU. These results indicate that WIK is a strain genetically less capable of swimming and that this motor trait is inherited to offspring.

## Discussion

In this study, we applied a swimmill and quantitatively analyzed swimming capability of zebrafish. First, we revealed that female and male fish have comparable capability in swimming. Second, we found a temperature range adequate for swimming. Third, we demonstrated that caudal fin length is related to swimming capability and that 3 mm length of caudal fin is sufficient for efficient swimming. Finally, we discovered that WIK strain is less capable of swimming compared to the other wild-type zebrafish strains. Interestingly, this motor trait was inherited to offspring.

It has been suggested that thermal conditions affect swimming capability of fish^13^. Indeed, many studies using large fish such as scombridae (*Scomber japonicus*) and tinfoil barbs (*Puntius schwanenfeldii*) have revealed that *U*_crit_ is compromised when assayed at lower water temperature^6,14,15^. Zebrafish is a freshwater tropical fish and is raised and maintained at 28.5 °C in laboratories^16^. Zebrafish is originated from India, Nepal and Bangladesh, where the temperature of river water is between 20 and 30 °C^17^. In this study, we measured *U*_crit_ at between 12 and 38 °C water temperature and unveiled that the adequate temperature for swimming was between 16 and 30 °C. It is reasonable that this range matches with wild condition, where zebrafish live. Interestingly, however, there is a report that a wild zebrafish was caught in a South Asian river where water temperature was above 38 °C^18^. As our study revealed that zebrafish cannot display good swimming performance at 38 °C, we assume that zebrafish in wild try to find deep and cool place in water when the water temperature goes above 30 °C. Zebrafish may also express certain proteins that allow them to cope with the elevated temperatures in hot summer.

Zebrafish gain driving force for forward movement by contracting caudal fin to left and right^7^. Since zebrafish can regenerate caudal fin if it was injured^19^, the caudal fin is presumably important for survival especially for escape from predators. A former study suggested that short-finned zebrafish is better at swimming compared to long-finned zebrafish^11^. We detailed the length of caudal fin in swimming performance and revealed that the adequate length of caudal fin was 3~10 mm. This is why the regular length of caudal fin is about 4~9 mm in short-finned zebrafish strains including AB and TU. On the other hand, long-finned zebrafish strains such as PET and TL carry a long caudal fin at the expense of lower swimming performance. Although we do not know whether long-finned zebrafish is found in wild, if so, why do they maintain such an extravagant trait? Handicap principle in ecology suggests that some bizarre and extravagant traits such as colorful tail of male peacock increase sex appeal and thus improve reproduction^20–23^. The long fin trait of zebrafish may also contribute to the reproduction. But, both female and male can have long fin in zebrafish and thus the handicap principle is inapplicable in this case.

Several zebrafish strains have been established and each strain has some unique traits. For example, the basal level of blood glucose in WIK is lower than that in TU^11^. Since WIK has a short fin and is rich in genetic heterogeneity, we originally assumed that WIK fish are good swimmer. Unexpectedly, however, WIK fish were worse swimmer even compared to the long-finned zebrafish. It is partly because WIK strain is originated from a single pair mating of wild zebrafish who might be less capable of swimming. As hybrid zebrafish raised by cross of TU and WIK strains showed high swimming capability and thus the low swimming performance in WIK is likely a recessive-type trait, the genomic region that is linked to the low swimming performance in WIK can be narrowed down in future studies.

## Materials and Methods

### Ethics statement

All animal experiments described in this manuscript and guidelines for use of zebrafish have been approved by Animal Care and Use Committee at Aoyama Gakuin University.

### Animals

Zebrafish (*Danio rerio*) were reared and maintained at 28 °C under a 14 h light and 10 h dark photoperiod and fed twice a day as in the regular care and maintenance protocol. We purchased four strains of adult zebrafish AB, Tübingen (TU), Tupfel long fin (TL) and Wild India Kolkata (WIK) from Zebrafish International Resource Center (ZIRC) located at the University of Oregon. The PET strain was obtained from a local pet distributor.

### Measurement of body length

The body length of adult zebrafish was measured using the Image J software (NIH) from movie frames of the swimming assay. The standard length (SL) and caudal fin length (CFL) were defined as the length from head to the root of the caudal fin and the caudal fin itself, respectively as described previously^7^.

### Swimming assay

We applied a Swim tunnel respirometer 170 mL (230 V/50 Hz; #SW10000) of Loligo System and measured critical swimming speed (*U*_crit_) as described previously^1^. Adult zebrafish of 6~9 months old were kept unfed for 24 h and used for the swimming assay. Briefly, zebrafish was placed in a glass chamber (length 11 cm, diameter 2.5 cm), compelled to swim in water flow and recorded by a high-speed video camera at 200 frames/s. Water flow was generated by spinning a propeller in the chamber. Water velocity was initially set at 10 cm/s for 1 min and then 15 cm/s for 1 min for warming up in our standard protocol. Since zebrafish swam against water flow to stay in the same position, they swam at the speed of water velocity. Eventually, the water velocity increased 1 cm/s every 1 min in a stepwise manner until the fish cannot keep swimming at the water velocity. The *U*_crit_ was calculated by an established equation^12^. *U*_crit_ = U + T/60. U (cm/s): the highest water velocity when zebrafish continued to swim for whole 1 min. T (s): time elapsed when fish can no longer keep swimming in 1 min. In a sudden swimming protocol, the initial water velocity was set at (*U*_crit_-3) cm/s followed by 1 cm/s increase every 1 min. Calibration of water velocity was done according to the manufacture instructions. In brief, we put fluorescent beads in the chamber, applied 0.5 to 4.5 V voltage to the motor of propeller and recorded the movement of fluorescent beads by the high-speed video camera. Using DPTV Flow Tracking System software (Loligo System), we monitored more than 30,000 fluorescent beads and calculated the water velocity. The water velocity was linear to the voltage throughout the entire range (Supplementary Fig. S1). The *U*_crit_ values of adult wild-type zebrafish in our study were comparable to those in the other study^24^. Note that the *U*_crit_ of zebrafish in the other study using a larger Swim tunnel system tends to show larger values^25^, but we did not figure this out why. In most assay, the water temperature was constantly kept at 26 ± 0.2 °C using a thermal heater. In some experiments, water temperature was increased by a thermal heater or decreased by putting an ice bag in the outside water tank. The dissolved oxygen concentration in the water before assay was 26~27%. All swimming assay were done in successive days. On day 0, fish were fed in the morning and then kept unfed for 24 h. On day 1, fish were used for swimming assay in late morning, fed after the assay and then kept unfed for 24 h. On day 2, fish were used for swimming assay in early afternoon.

### Fin clip

The PET strain zebrafish that have a long caudal fin (14~30 mm) were purchased from a local pet shop and used to investigate the effect of caudal fin length in swimming capability. They were subjected to swimming assay as intact on day 1 and 2. After swimming assay on day 2, caudal fin were clipped to 10 mm. They were subjected to swimming assay on day 3 and 4 and their caudal fin were cut to 5 mm. They were then subjected to swimming assay on day 5 and 6 and their caudal fin were further shortened to 3 mm. They were assayed for swimming on day 7 and 8 and their caudal fin were further eliminated to 1 mm. The final swimming assay was done on day 9 and 10. The *U*_crit_ on even numbered days were used for analysis. In this multi-day fin clip experiments, fin was clipped just before feeding and kept unfed for about 24 h before swimming assay.

## Supplementary information

Supplemental information

## References

[1] Conradsen C, Walker JA, Perna C, Mcguigan K (2016). Repeatability of locomotor performance and morphology–locomotor performance relationships. Journal of Experimental Biology.

[2] Widrick JJ (2018). An open source microcontroller based flume for evaluating swimming performance of larval, juvenile, and adult zebrafish. PLoS One.

[3] Kolok AS (1991). Photoperiod alters the critical swimming speed of juvenile largemouth bass, Micropterus salmoides, acclimated to cold water. Copeia.

[4] Conradsen C, Mcguigan K (2015). Sexually dimorphic morphology and swimming performance relationships in wild-type zebrafish Danio rerio. Journal of fish biology.

[5] Leris I, Sfakianakis DG, Kentouri M (2013). Are zebrafish Danio rerio males better swimmers than females?. Journal of Fish Biology.

[6] Peake S, Mckinley R, Barth C (1997). Effect of recovery parameters on critical swimming speed of juvenile rainbow trout (Oncorhynchus mykiss). Canadian Journal of Zoology.

[7] Plaut I (2000). Effects of fin size on swimming performance, swimming behaviour and routine activity of zebrafish Danio rerio. Journal of Experimental Biology.

[8] The Zebrafish Information Network (ZFIN), https://zfin.org/.

[9] Gorissen M (2015). Differences in inhibitory avoidance, cortisol and brain gene expression in TL and AB zebrafish. Genes, Brain and Behavior.

[10] Van Den Bos R (2019). Early life exposure to cortisol in zebrafish (Danio rerio): similarities and differences in behaviour and physiology between larvae of the AB and TL strains. Behavioural pharmacology.

[11] Meyer BM, Froehlich JM, Galt NJ, Biga PR (2013). Inbred strains of zebrafish exhibit variation in growth performance and myostatin expression following fasting. Comparative Biochemistry and Physiology Part A: Molecular & Integrative Physiology.

[12] Brett JR (1964). The respiratory metabolism and swimming performance of young sockeye salmon. Journal of the Fisheries Board of Canada.

[13] Randall D, Brauner C (1991). Effects of environmental on exercise in fish. Journal of Experiental Biology.

[14] O’steen S, Bennett AF (2003). Thermal acclimation effects differ between voluntary, maximum, and critical swimming velocities in two cyprinid fishes. Physiological and Biochemical Zoology.

[15] Dickson KA, Donley JM, Sepulveda C, Bhoopat L (2002). Effects of temperature on sustained swimming performance and swimming kinematics of the chub mackerel Scomber japonicus. Journal of experimental biology.

[16] Kimmel CB (1995). Stages of embryonic development of the zebrafish. Developmental dynamics.

[17] Weather to visit. Supaul monthly weather averages. *figshare*, https://www.weather2visit.com/asia/india/supaul.htm (2019)

[18] Engeszer RE, Patteroson LB, Rao AA, Parichy DM (2007). Zebrafish in the wild: a review of natural history and new notes from the field. Zebrafish.

[19] Poss KD, Keating MT, Nechiporuk A (2003). Tales of regeneration in zebrafish. Developmental dynamics: an official publication of the American Association of Anatomists.

[20] Biernaskie JM, Perry JC, Grafen A (2018). A general model of biological signals, from cues to handicaps. Evolution Letters.

[21] Grafen A (1990). Sexual selection unhandicapped by the Fisher process. Journal of theoretical biology.

[22] Grafen A (1990). Biological signals as handicaps. Journal of theoretical biology.

[23] Zahavi A (1975). Mate selection—a selection for a handicap. Journal of theoretical Biology.

[24] Parisi, A. PGC1α and exercise adaptations in zebrafish. BioRxiv, 10.1101/483784 (2018).

[25] Makalled MH (2016). Injury-induced ctgfa directs glial bridging and spinal cord regeneration in zebrafish. Science.

