# Supplementary figures and images for: Swimming capability of zebrafish is governed by water temperature, caudal fin length and genetic background
Source: Sci Rep. 2019 Nov 8;9:16307. doi: 10.1038/s41598-019-52592-w (PMC6841939; doi:10.1038/s41598-019-52592-w)

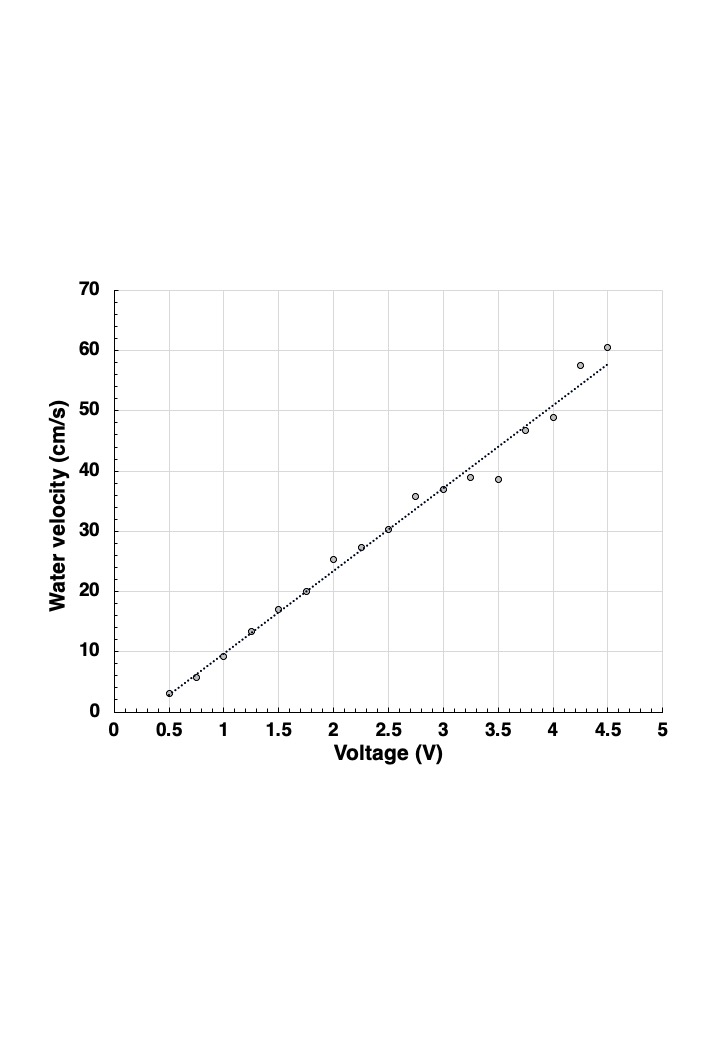

Supplement: Supplementary file 1 — Supplemental information [file 41598_2019_52592_MOESM1_ESM.jpeg]
